# A Cascade of Thermophilic Enzymes As an Approach to the Synthesis of Modified Nucleotides

**Published:** 2016

**Authors:** R. S. Esipov, Yu. A. Abramchik, I. V. Fateev, I. D. Konstantinova, M. A. Kostromina, T. I. Muravyova, K. G. Artemova, A. I. Miroshnikov

**Affiliations:** Shemyakin and Ovchinnikov Institute of Bioorganic Chemistry, Miklukho-Maklaya Str., 16/10, Moscow, GSP-7, 117997, Russia

**Keywords:** enzymatic nucleotide synthesis, ribokinase, phosphoribosylpyrophosphate synthetase, adenine phosphoribosyltransferase, thermophilic microorganisms, substrate properties

## Abstract

We propose a new approach for the synthesis of biologically important nucleotides which includes a multi-enzymatic cascade conversion of *D*-pentoses into purine nucleotides. The approach exploits nucleic acid exchange enzymes from thermophilic microorganisms: ribokinase, phosphoribosylpyrophosphate synthetase, and adenine phosphoribosyltransferase. We cloned the ribokinase gene from *Thermus sp*. 2.9, as well as two different genes of phosphoribosylpyrophosphate synthetase (PRPP-synthetase) and the adenine phosphoribosyltransferase (APR-transferase) gene from *Thermus thermophilus *HB27 into the expression vectors, generated high-yield *E. coli *producer strains, developed methods for the purification of the enzymes, and investigated enzyme substrate specificity. The enzymes were used for the conversion of *D*-pentoses into 5-phosphates that were further converted into 5-phospho-α-*D*-pentofuranose 1-pyrophosphates by means of ribokinase and PRPP-synthetases. Target nucleotides were obtained through the condensation of the pyrophosphates with adenine and its derivatives in a reaction catalyzed by APR-transferase. 2-Chloro- and 2-fluoroadenosine monophosphates were synthesized from *D*-ribose and appropriate heterobases in one pot using a system of thermophilic enzymes in the presence of ATP, ribokinase, PRPP-synthetase, and APR-transferase.

## INTRODUCTION

5’-phosphorylated nucleosides are important metabolites of DNA and RNA biosynthesis, as well as co-substrates and cofactors of numerous biochemical reactions [1-3]. The important role these compounds play in a living cell underlies the interest in the synthesis of not only natural members of this class, but also their various analogues to directly affect the metabolism in normal and pathological conditions [4-8]. A large number of heterobase- and sugar-modified nucleosides are used as important antiviral and anticancer agents [9-13]. The effect of modified nucleosides is mediated through their intracellular conversion primarily into 5’-monophosphates and then usually into 5’-di- and triphosphates that act as antimetabolites. The first step in the metabolic activation of modified nucleosides – conversion into 5’-monophosphates – is known to determine the nucleoside’s biological properties. It should also be noted that heterobase- and/or sugar-modified nucleoside-5’-monophosphates are of considerable interest as starting materials for the chemical synthesis of phosphate derivatives (prodrugs) and enzymatic conversion into 5’-triphosphates for subsequent inclusion in oligonucleotides [14-16]. The development of effective biosynthetic approaches to the production of 5’-monophosphates of modified nucleosides draws the attention of researchers engaged in the development of highly efficient chemotherapeutic agents.

**Fig. 1:**
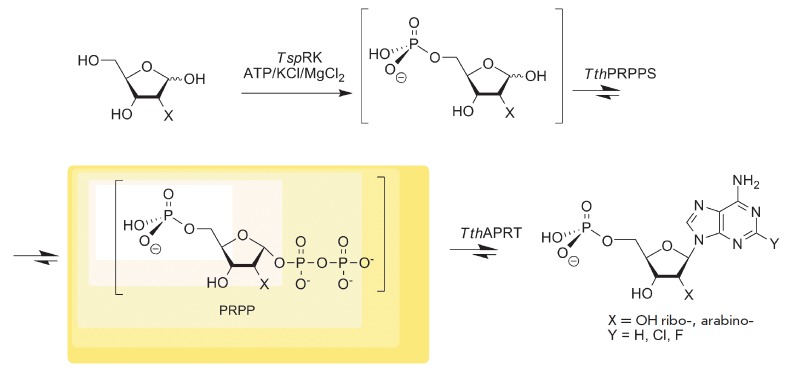
A diagram of the multi-enzymatic cascade synthesis of substituted adenosine-5’-monophosphates.

Mono- and multi-enzymatic synthesis of nucleoside 5’-mono- and 5’-triphosphates has been the subject of numerous publications [17-21]. Of these, we were particularly interested in the phosphoribosyltransferases that were recently successfully used in a cascade five-component synthesis of purine riboside-5’-monophosphates [22-24]. Nucleoside phosphorylases of thermophilic microorganisms are known to be less sensitive to the substrate structure [25, 26], which enables them to act at 70–80°C and significantly increases the enzymatic reaction efficiency due to the increased solubility of heterocyclic substrates [27]. All these data aroused our interest in the purification of the recombinant enzymes ribokinase, phosphoribosylpyrophosphate synthetase, and adenine phosphoribosyltransferase from thermophilic microorganisms and in the investigation of their substrate properties to determine the applicability of these enzymes in the cascade synthesis of purine nucleoside-5-monophosphates according to the Diagram in *Fig. 1*.

## MATERIALS AND METHODS

**Cloning**

Genes TT_RS05985, TT_RS06430, and TT_RS06315 encoding *Tth*PRPPS1, *Tth*PRPPS2, and *Tth*APRT, respectively, were amplified on the genomic DNA template of the *Thermus thermophilus *HB27 strain by a polymerase chain reaction (PCR) using synthetic primers. The QT17_05185 gene encoding RK from *Thermus *sp. 2.9 was codon-optimized for expression in *Escherichia coli *and synthesized by a chemical and enzymatic method from overlapping oligonucleotides. All genes were cloned into the expression vector pET-23d+ at the NcoI and XhoI restriction endonuclease recognition sites.

**Cultivation of producer strains**

*E. coli *BL21(DE3), Rosetta (DE3), and C3029/pGTf2 strains were transformed with the produced expression vectors pER-PRPPS1-Tth, pER-PRPPS2-Tth, pER-APRT-Tth, and pER-RK-Tsp. Producer strains derived from *E. coli *BL21(DE3) and Rosetta(DE3) were cultured at 37 °C in a LB medium containing ampicillin (100 μg/mL). Cultivation of the producer strains derived from *E. coli *C3029/pGTf2 was carried out in a LB medium containing 50 μg/mL ampicillin, 20 μg/mL chloramphenicol, and 1 ng/mL tetracycline. After reaching a absorbance of A_595_ = 0.8, the cultures were added with IPTG to a final concentration of 0.4 mM and cultivation was continued at 23 and 37 °C. The cultivation duration varied from 4 to 16 h, depending on the strain. After culturing, the cell biomass was separated by centrifugation, homogenized at a 1:10 (w/v) ratio in a buffer solution (50 mM Tris-HCl, pH 8.5, 150 mM NaCl, 2 mM PMSF), and disrupted using a Labsonic P ultrasonicator (Sartorius, Germany) at 4°C for 10 min (cycle, 0.4 sec; amplitude, 30%). The amount of target enzymes in soluble and insoluble cell fractions was determined by densitometric analysis of electrophoretic gels using the ImageLab 5.0 software (Bio-Rad, USA) [28]. The producer strains containing the maximum amount of a targeted protein in the supernatant were chosen: *E. coli *BL21(DE3)/pER-APRT-Tth (culturing at 37 °C for 4 h after adding IPTG), *E. coli *Rosetta(DE3)/pER-PRPPS1-Tth (37 °C for 4 h), *E. coli *S3029/pGTf2/pER-PRPPS2-Tth (37 °C for 5 h), and *E. coli *S3029/pGTf2/pER-RK-Tsp (23 °C for 16 h). The strains were grown in 5–6 L of the culture medium.

**Isolation and purification of TthPRPPS1,**

Cell biomass of the producer strains *Tth*PRPPS1, *Tth*PRPPS2, and *Tsp*RK was re-suspended in a buffer solution (50 mM Tris-HCl, pH 8.7, 1 mM PMSF) at a 1:10 (w/v) ratio and disrupted using the Labsonic P ultrasonicator at +4 °C for 20 min (cycle, 0.5 sec; amplitude, 50%). Cell debris was removed by centrifugation at 12,000 rpm at +4 °C for 30 min on a Hermle Z383K centrifuge (HERMLE Labortechnik GmbH, Germany). In purification of *Tsp*RK, a clarified cell lysate was heat-treated at 65 °C for 10 min to precipitate contaminating proteins and DNA. The precipitate was removed by centrifugation. Further purification of the enzymes was carried out according to the same scheme. A clarified cell lysate was loaded onto a XK 16/20 column (GE Healthcare, USA) with Ni^2+^-IDA resin (Qiagen, Germany) pre-equilibrated with 50 mM Tris-HCl buffer, pH 8.7. Ballast proteins were removed by washing with 50 mM Tris-HCl buffer, pH 8.7, containing 50 mM imidazole. A target protein was eluted with a buffer solution of 50 mM Tris-HCl and 200 mM imidazole, pH 8.7. After affinity chromatography, fractions containing a target protein were added with EDTA to a concentration of 5 mM and concentrated using an Amicon 8200 200 mL stirred ultrafiltration cell (Millipore, USA) on an YM 10 kDa membrane (Millipore) when purified *Tth*PRPPS1 and *Tth*PRPPS2 and on an YM 30 kDa membrane (Millipore) when purified *Tsp*RK. Further purification was performed on a HiLoad 16/60 column with Superdex 200 resin (GE Healthcare) equilibrated with buffer containing 20 mM Tris-HCl, 1 mM ATP, 1 mM MgCl_2_, 5% glycerol, 0.04% NaN_3_, pH 8.5. Fractions containing a target protein were pooled and concentrated by ultrafiltration to a final concentration of 12 ± 1 mg/mL as previously described. The protein concentration was determined by the Bradford method using BSA as a standard [29]. Protein purity was determined by polyacrylamide gel electrophoresis under denaturing conditions [28]. Purified enzymes were stored at –80 °C.

**Isolation and purification of TthAPRT**

Cell biomass of the *Tth*APRT producer strain was disrupted according to the procedure described for the other enzymes. A clarified cell lysate was added with NaCl to a concentration of 300 mM and heat-treated at 65 °C for 10 min. After ballast protein precipitation by centrifugation, the lysate was applied to a PD-10 column with Sephadex G-25 Medium resin (GE Healthcare, USA) equilibrated with a buffer solution containing 20 mM Tris-HCl and 1.0 mM EDTA, pH 9.0. After desalting, the protein solution was loaded onto a XK 16/20 column with Q Sepharose XL (GE Healthcare, USA) equilibrated with the same buffer solution. A target protein was eluted with a linear concentration gradient of NaCl (0 to 400 mM). Fractions containing the target protein were pooled and loaded onto an XK 16/20 column with Phenyl Sepharose HP (GE Healthcare, USA) equilibrated with buffer containing 20 mM Tris-HCl, 1 M (NH_4_)_2_SO_4_, 1.0 mM EDTA, pH 7.6. *Tth*APRT was eluted with a linear gradient of (NH_4_)_2_SO_4_ (from 1 to 0 M). Fractions containing the target protein were pooled and concentrated by ultrafiltration on a PBGC 10 kDa polysulfone membrane to a final concentration of 5.0 ± 0.5 mg/mL as previously described. The final purification was performed on a HiLoad 16/60 column with Superdex 200 resin equilibrated with 20 mM Tris-HCl buffer, pH 8.0, containing 50 mM NaCl, 5% glycerol, and 0.04% NaN_3_. Fractions containing the target protein were pooled and concentrated by ultrafiltration to a final concentration of 12 ± 1 mg/mL. The protein purity and concentration were determined as described previously [28, 29]. The purified enzyme was stored at –80 °C.

**Enzymatic activity assay**

The *Tsp*RK activity was determined radiochemically based on the formation of *D*-ribofuranosyl-5-[32P]phosphate in the presence of [γ-^32^P]ATP. A reaction mixture (0.05 mL, 20 mM Tris-HCl, pH 8.0) contained a 0.4 mM disodium ATP salt, 1 mM *D*-ribose, 5 mM MgCl_2_, 50 mM KCl, 1 mM KH_2_PO_4_, 6 μCi of [γ-^32^P]ATP, and 0.15 μg of *Tsp*RK. The mixture was incubated at 75 °C. Then, 0.8 μL aliquots were taken at 10, 20, and 40 min, applied to plates with PEI-cellulose, and eluted with 0.5 M aqueous potassium dihydrogen orthophosphate. The amount of *D*-ribofuranosyl-5-[^32^P]phosphate was determined on a TRI-CARB 2100TR liquid scintillation counter (Packard BioScience Co.).

The *Tth*PRPPS1 and *Tth*PRPPS2 activity was determined in a reaction mixture containing a 1 mM disodium ATP salt, a 1 mM disodium salt of *D*-ribose-5 phosphate, 5 mM MgCl_2_, 10 mM KH_2_PO_4_, 20 mM Tris- HCl, pH 8.0, at 75 °C. *Tth*PRPPS (0.75 μg) was added to 0.5 mL of the mixture.

The *Tth*APRT activity was determined in a reaction mixture containing 1 mM adenine, a 1 mM pentasodium salt of 5-phosphoribosyl-α-1-pyrophosphate (PRPP), 5 mM MgCl_2_, and 20 mM Tris-HCl, pH 8.0, at 75 °C. *Tth*APRT (0.125 μg) was added to 0.5 mL of the reaction mixture. The amount of the product (μM) formed for 1 min was taken as the activity unit.

**Determination of ribokinase kinetic parameters**

A reaction mixture (0.5 mL, 20 mM Tris-HCl, pH 8.0) contained (a) a disodium salt of ATP (0.01 to 0.6 mM) and *D*-ribose (1 mM) or (b) *D*-ribose (0.01 to 8.0 mM) and ATP (1 mM) to determine the *K*_M_ and *V*_max_ values for ATP and *D*-ribose, respectively, 5 mM MgCl_2_, 50 mM KCl, 10 mM KH_2_PO_4_, and 0.0175 μg of *Tsp*RK. Each experiment used 16 reaction mixtures. The mixtures were incubated at 75 °C for 12 min; each experiment was performed in triplicate. Then, the mean rate was determined in three experiments at the same enzyme concentration. Substrate and product concentrations were determined by HPLC under isocratic elution with 0.1 M KH_2_PO_4_ (water, pH 6.0, flow rate of 0.5 mL/min) with detection at 254 nm (Waters 2489 UV detector; Supelcosil LC-18-T column, 5 μm, 150 × 4.6 mm).

**Determination of the kinetic parameters of PRPP-synthetases**

The kinetic parameters of the enzymes were determined at a ATP concentration that varied in the range of 0.005 to 0.2 mM. A similar interval was used for *D*-ribose-5 phosphate. The remaining conditions were the same as those in the enzymatic activity assay. The reaction was conducted for 2 min in triplicate. Then, the mean rate was determined in three experiments at the same concentration. Concentrations of ATP and AMP were determined by HPLC under isocratic elution with 0.1 M KH_2_PO_4_ (pH 6.0, flow rate of 0.5 mL/min) with detection at 254 nm (Waters 2489 detector; Supelcosil LC-18-T column, 5 μm, 150 × 4.6 mm).

**Determination of the kinetic parameters of APR-transferase**

The kinetic parameters of APR-transferase were determined at an adenine concentration that varied in the range of 0.005 to 0.2 mM and a PRPP concentration that varied from 0.05 to 1.2 mM. The remaining conditions were the same as in the enzymatic activity assay. The reaction was conducted for 1 min in triplicate. The mean reaction rate was determined based on the results of three experiments at the same concentration. Adenine and AMP concentrations were determined by HPLC under isocratic elution with 36% aqueous methanol (flow rate of 0.5 mL/min) with detection at 254 nm (Waters 2489 detector; MZ PerfectSil 100 ODS-3 column, 5 μm, 150 × 4.6 mm).

The kinetic parameters were determined by a nonlinear regression analysis using the SciDAVis v.0.2.4 software. The apparent catalytic constant (k_cat_) was calculated per one subunit, whose weight was determined based on the amino acid sequence (32.0 kDa for *Tsp*RK, 34.5 kDa for *Tth*PRPPS1, 34.6 kDa for *Tth*PRPPS2, and 19.0 kDa for *Tth*APRT).

## RESULTS AND DISCUSSION

To study the possibility of three-step nucleotide biosynthesis, we carried out comprehensive work on the production of recombinant enzymes of the nucleic acid metabolism (ribokinase, two PRPP-synthetases, and APR-transferase from *T. thermophilus*) and an investigation of their substrate properties

The QT17_05185 gene encoding RK from *Thermus *sp. 2.9 was generated by a chemical-enzymatic method. Genes TT_RS05985, TT_RS06430, and TT_RS06315 encoding *Tth*PRPPS1, *Tth*PRPPS2, and *Tth*APRT from *T. thermophilus *HB27, respectively, were amplified from the genomic DNA by PCR. All genes were cloned into the plasmid vector pET23d+. The resulting expression vectors pER-PRPPS1-Tth, pER-PRPPS2- Tth, and pER-RK-Tsp contained hybrid genes with a reading frame including sequences encoding enzymes and an affinity tag of six histidine residues. The expression vector pER-APRT1-Tth contained an unmodified sequence encoding *Tth*APRT.

The plasmids were used to transform the *E. coli *strains BL21(DE3), Rosetta(DE3), and C3029/pGTf2. We determined the culture conditions under which producer strains synthesized target enzymes in soluble form. Producers of soluble recombinant *Tsp*RK and *Tth*PRPPS2 were derived from *E. coli *S3029/pGTf2. This strain was generated by transforming *E. coli *S3029 cells with the plasmid vector pGTf2 (Takara Bio Inc) carrying sequences encoding GroES-GroEL-Tig chaperones under the control of the Pzt-1 promoter. The choice of the *E. coli *Rosetta(DE3) strain for *Tth*PRPPS1 biosynthesis was based on the fact that the TT_RS05985 gene encoding *Tth*PRPPS1 contains codons rarely used in *E. coli *(one AGA, nine AGGs, 10 CGGs, one AUA, one CUA, and 15 CCCs). The Rosetta(DE) strain synthesizes tRNAs for these rare codons. Soluble recombinant *Tth*APRT was produced in the *E. coli *BL21(DE3) strain.

Isolation and purification of ribokinase and two PRPP-synthetases included metal chelate and gel filtration chromatography stages (*Table 1*). Heat treatment of the cell lysate during *Tsp*RK purification significantly enriched the target protein fraction due to aggregation of cellular proteins and DNA. A similar treatment of both *Tth*PRPP-synthetases did not provide positive results, but it led to a loss of the target protein. Due to the high probability of proteolysis, metal chelate chromatography was conducted under cooling conditions, with addition of EDTA to the pooled fractions. Following the final purification step using gel filtration chromatography, all protein samples were concentrated.

Isolation and purification of *Tth*APRT included heat treatment, desalting, concentrating, and anion exchange, hydrophobic interaction, and gel filtration chromatography steps (*Table 1*). As in the case of *Tsp*RK, the first step involved heat treatment of a clarified cell lysate with addition of a salt to the solution (up to 300 mM sodium chloride), which greatly accelerated the aggregation of contaminating cell proteins and DNA.

**Table 1:** Steps of the isolation and purification of ribokinase, PRPP-synthetases, and APR-transferase

Purification step	Volume, mL	Protein concentration, mg/mL	Total protein, mg
Ribokinase from Thermus sp. 2.9			
1. Ultrasonic disintegration	150*	8.3	1245
2. Heat treatment	136	1.7	231.2
3. Metal chelate chromatography	30	1.9	57
4. Concentrating	9	6	54
5. Gel filtration chromatography	28.5	1.28	36.4
6. Concentrating	2.6	11.7	30.4
PRPP-synthetase 1 from T. thermophilus HB27			
1. Ultrasonic disintegration	150**	10	1500
2. Affinity chromatography	100	0.8	80
3. Concentrating	13	5.7	74.1
4. Gel filtration	50	1	50
5. Concentrating	4	12.3	49.2
PRPP-synthetase 2 from T. thermophilus HB27			
1. Ultrasonic disintegration	130**	11.2	1456
2. Affinity chromatography	125	0.6	75
3. Concentrating	10.3	6.2	63.9
4. Gel filtration	37.5	1.2	45
5. Concentrating	3.4	12	40.8
APR-transferase from T. thermophilus HB27			
1. Ultrasonic disintegration	157***	6	942
2. Heat treatment	149	1.3	193.7
3. Gel filtration	258	0.7	181
4. Anion exchange chromatography	72	1.2	86.4
5. Hydrophobic interaction chromatography	73	0.8	58.4
6. Concentrating	11	5	55
7. Gel filtration chromatography	52	0.87	45.2
8. Concentrating	3.4	12.5	42.5

^*^From 5.8 L of culture.

^**^From 5 L of culture.

^***^From 6 L of culture.

The developed techniques provided a yield of all recombinant thermophilic enzymes not less than 8–10 mg per 1 L of culture medium, with an electrophoretic purity of at least 95%.

Next, we determined the kinetic parameters and optimal activity conditions for the purified recombinant enzymes.

The specific activity of *Tsp*RK was determined by a radiochemical method based on the formation of *D*-ribofuranosyl-5-[^32^P]phosphate in the presence of [γ-^32^P] ATP. The amount of the product (μM) formed for 1 min was taken as the activity unit. The *Tsp*RK activity was 5.5 U/mg.

The specific activity of *Tth*PRPPS1 and *Tth*PRPPS2 was determined indirectly via AMP formation in a reaction mixture (according to HPLC) in the presence of two substrates: ATP and *D*-ribose-5-phosphate. The activity was 0.85 U/mg for the former enzyme and 11 U/mg for the latter enzyme. Given the large differences in the enzyme activity, using the second synthetase for nucleotide synthesis seems to be more rational, with allowance for lower protein consumption. The *Tth*APRT properties were studied in a model reaction of adenine with 5-phosphoribosyl-α-1-pyrophosphate. The AMP formation was monitored by HPLC. The specific enzyme activity was 8.8 U/mg.

A high concentration of ribose as a substrate was found to inhibit the enzymatic activity of *Tsp*RK. This effect was described for human ribokinase [30]. Therefore, the results of ATP experiments were analyzed using the Michaelis-Menten equation *V *= *V*_max_ × *S */ (*K*_M_ + *S*). In experiments with *D*-ribose, we used an equation allowing for the substrate inhibition effect due to the binding of a second molecule *V *= *V*_max_ × *S */ (*K*_M_ + *S *+ *S*^2^ / *K*_i_).

**Table 2:** Kinetic parameters of the natural substrates of the studied enzymes

Substrate	K_M_, μM	K_i_, μM	V_max_, μM/min·mg	k_cat_, 1/s	k_cat_/K_M_, 1/M·s
Ribokinase from Thermus sp. 2.9					
ATP	75 ± 11	–	13 ± 1	6.8 ± 0.7	9.1 × 10^4^
D-ribose	20 ± 6	1,700 ± 400	13 ± 2	7.1 ± 1.2	3.5 × 10^5^
PRPP-synthetase 1 from T. thermophilus HB27					
ATP	10 ± 2	–	0.71 ± 0.05	0.41 ± 0.03	4.3 × 10^4^
D-ribose-5 phosphate	32 ± 6	–	0.85 ± 0.11	0.49 ± 0.06	1.5 × 10^4^
PRPP-synthetase 2 from T. thermophilus HB27					
ATP	12 ± 2	–	20 ± 2	11 ± 1	9.9 × 10^5^
D-ribose-5 phosphate	40 ± 4	–	24 ± 2	14 ± 1	3.4 × 10^5^
APR-transferase from T. thermophilus HB27					
Adenine	13 ± 2	–	6.0 ± 0.4	1.9 ± 0.1	1.4 × 10^5^
PRPP	179 ± 35	–	9.2 ± 1.1	2.9 ± 0.4	1.6 × 10^4^

The kinetic parameters of natural substrates of the enzymes are listed in *Table 2*.

**Fig. 2:**
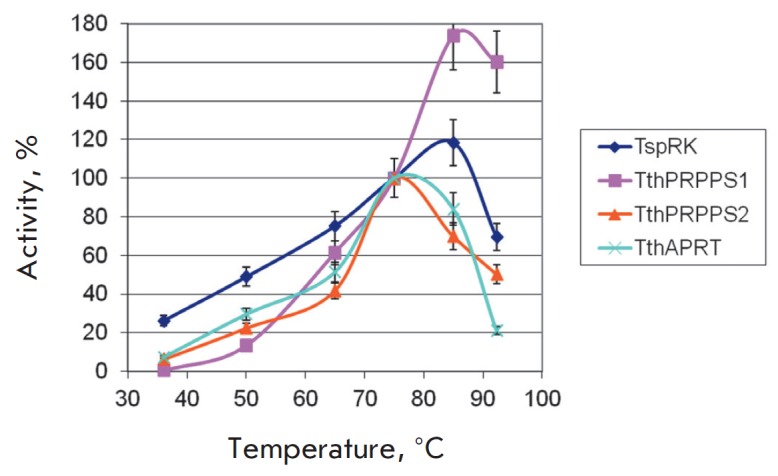
The temperature dependence of the enzyme activity. The activity at 75 °C was taken as 100%. Reactions were conducted in a temperature range from 36 to 92 °C in 0.05 mL of 20 mM Tris-HCl buffer, pH 8.0, containing: 1) 0.4 mM ATP, 1 mM ribose, 1 mM KH_2_PO_4_, 5 mM MgCl_2_, 50 mM KCl, 0.15 μg of *Tsp*RK, 2) 1 mM ATP, 1 mM *D*-ribose 5-phosphate, 5 mM MgCl_2_, 10 mM KH_2_PO_4_, 0.75 μg of *Tth*PRPPS1 or *Tth*PRPPS2, 3) 1 mM adenine, 1 mM PRPP, 5 mM MgCl_2_, 0.125 μg of *Tth*APRT.

**Fig. 3:**
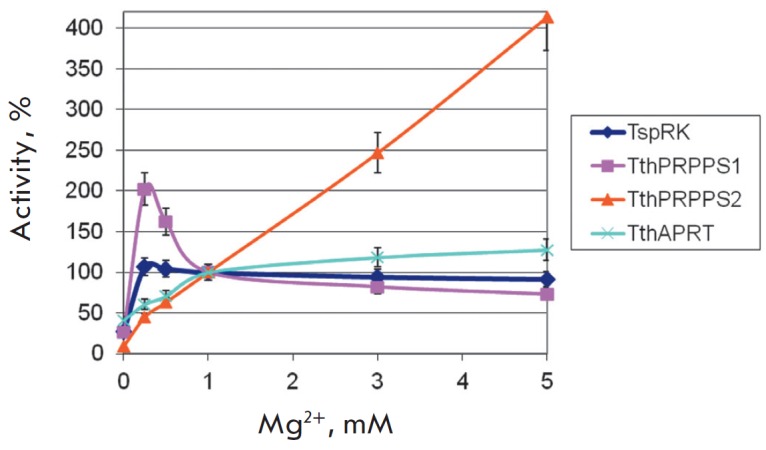
The dependence of enzyme activity on the magnesium ion concentration. The activity in the presence of 1 mM Mg^2+^ was taken as 100%. Reactions were conducted at a temperature of 75°C, at a MgCl_2_ concentration that varied from 0 to 5 mM, in 0.5 mL of 20 mM Tris-HCl buffer, pH 8.0, containing: 1) 0.4 mM ATP, 1 mM ribose, 1 mM KH_2_PO_4_, 50 mM KCl, 0.15 μg of *Tsp*RK, 2) 1 mM ATP, 1 mM *D*-ribose 5-phosphate, 10 mM KH_2_PO_4_, 0.75 μg of *Tth*PRPPS1 or *Tth*PRPPS2, 3) 1 mM adenine, 1 mM PRPP, 0.125 μg of *Tth*APRT.

**Fig. 4:**
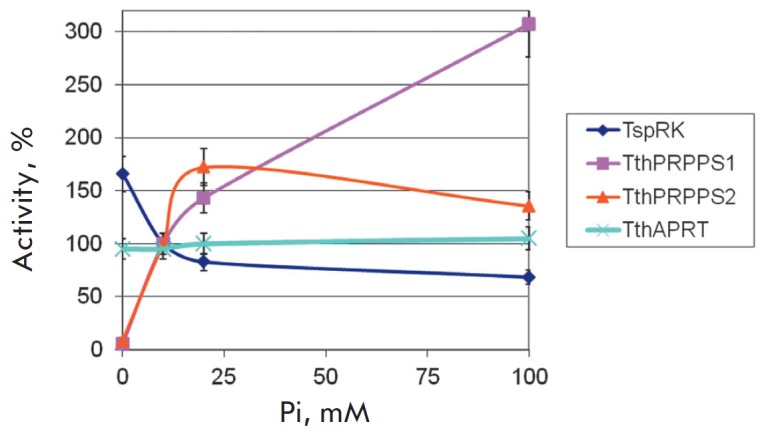
The dependence of enzyme activity on the inorganic phosphate concentration. The activity in the presence of 10 mM Pi was taken as 100%. Reactions were conducted at a temperature of 75°C, at a KH_2_PO_4_ concentration that varied from 0 to 100 mM, in 0.5 mL of 20 mM Tris-HCl buffer, pH 8.0, 5 mM MgCl_2_, containing: 1) 0.4 mM ATP, 1 mM ribose, 50 mM KCl, 0.15 μg of *Tsp*RK, 2) 1 mM ATP, 1 mM *D*-ribose 5-phosphate, 0.75 μg of *Tth*PRPPS1 or *Tth*PRPPS2, 3) 1 mM adenine, 1 mM PRPP, 0.125 μg of *Tth*APRT.

*Figure 2*, *Figure 3*, *Figure 4* present the results of experiments on exploring optimal conditions for the activity of the recombinant enzymes.

The studied enzymes were active in a broad temperature range. The maximum activity of *Tsp*RK and *Tth*PRPPS1 was observed at 85°C, whereas the *Tth*APRT activity at this temperature was lower by 35%. Therefore, we decided to conduct further cascade syntheses at 75°C.

The activity of the enzymes in a buffer solution lacking magnesium ions was very low. Addition of magnesium chloride to a concentration of 0.25 mM led to a considerable increase in the activity.

At higher concentrations, the dependence of enzyme activity on the magnesium ion concentration was as follows: the activity decreased in the case of *Tsp*RK and *Tth*PRPPS1, increased in *Tth*APRT, and drastically increased in *Tth*PRPPS2. Thus, the cascade reactions involving *Tth*PRPPS1 should be conducted at a magnesium chloride concentration of 0.25–0.5 mM; and those involving *Tth*PRPPS2 – at 5 mM magnesium chloride.

The enzymes responded differently to the addition of potassium orthophosphate. The activity decreased in the case of *Tsp*RK, remained unchanged in *Tth*APRT, and significantly increased in both *Tth*PRPPSs, with the *Tth*PRPPS2 activity starting to decrease at a salt concentration of more than 25 mM. The optimum Pi concentration for conducting cascade reactions is 10– 25 mM.

The most crucial question remained the substrate specificity of each enzyme for different carbohydrates.

**Fig. 5:**
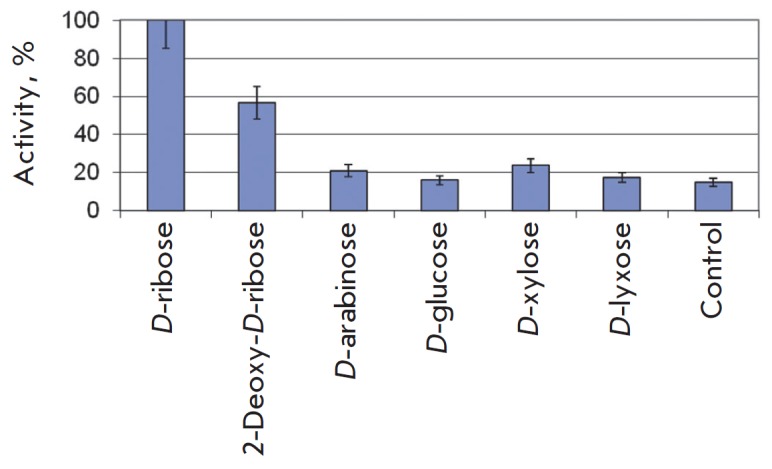
Enzymatic activity of ribokinase in the presence of different carbohydrates. Reactions were conducted at a temperature of 75°C in 0.05 mL of 20 mM Tris-HCl buffer, pH 8.0, containing 0.4 mM ATP, 1 mM carbohydrate (no carbohydrates in the control reaction), 1 mM KH_2_PO_4_, 5 mM MgCl_2_, 50 mM KCl, 0.15 μg of RK.

*D*-ribose and 2-deoxy-*D*-ribose are *Tsp*RK substrates (*Fig. 5*), with the activity toward deoxy-sugar being 57% of that for *D*-ribose. The activity toward *D*-arabinose and *D*-xylose was 2 times higher than the background activity, which may indicate that they can be used as substrates, but much less specific ones. Activity for *D*-glucose and *D*-lyxose was not detected.

**Fig. 6:**
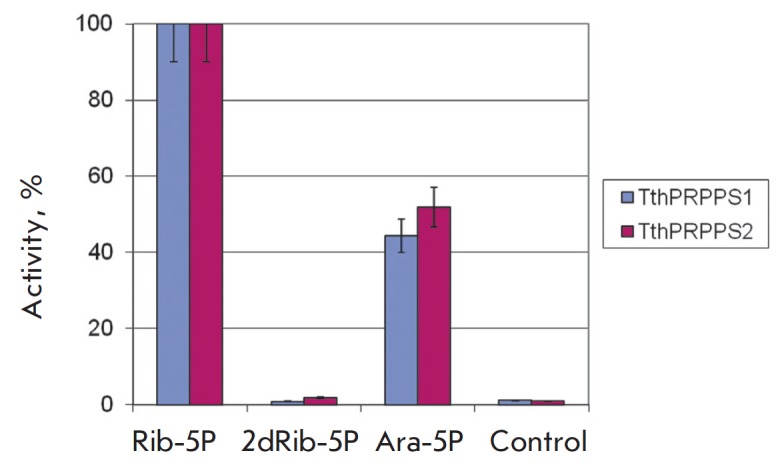
Substrate specificity of PRPP synthetases toward various carbohydrate-5-phosphates. Reactions were conducted at a temperature of 75 °C in 0.5 mL of 20 mM Tris-HCl buffer, pH 8.0, containing 1 mM ATP, 1 mM carbohydrate-5-phosphate (no carbohydrates in the control reaction), 5 mM MgCl_2_, 10 mM KH_2_PO_4_, 0.75 μg of PRPPS.

We tested the activity of both *Tth*PRPPSs toward 2’-deoxy-ATP, GTP, and 2’-deoxy-GTP. The activity for 2’-deoxy-ATP was 80% of that for ATP. No activity toward GTP and 2’-deoxy-GTP was detected (data not shown). It may be that these *Tth*PRPPSs are first-type enzymes [31]. All reactions were conducted under the same conditions as those for the activity assay: only the added substrate was varied. Both enzymes were able to catalyze the reaction with *D*-arabinose-5-phosphate (*Fig. 6*). However, activity for 2-deoxy-*D*-ribose-5 phosphate was not detected, indicating the importance of a hydroxyl group at the second sugar position to the enzymatic reaction.

**Table 3:** Substrates of APR-transferase

Base	Conversion for 24 h, %	MS of product, [M+H]^+^
2,6-Diaminopurine	16.8	363.0786 (calc. 363.0813)
2-Chloroadenine	97.6	382.0257 (calc. 382.0314)
2-Fluoroadenine	36.5	366.0564 (calc. 366.0611)
Adenine	50.0	348.0677 (calc. 348.0704)
2-Methoxyadenine	60.9	378.0812 (calc. 378.0809)
N1-methyladenine	78.2	362.0843 (calc. 362.0800)
N6-benzyladenine	1.9	438.1117 (calc. 438.1173)
2-Aminobenzimidazole	0.1	346.0796 (calc. 346.0799)
1,2,4-Triazole-3-carboxy-N-methylamide	0	–
Guanine	0	–
Hypoxanthine	0	–
7-Deaza-2,6-diaminopurine	0	–

Note. Reaction conditions: reaction mixtures (0.5 mL; 20 mM Tris-HCl, pH 8.0, 75 °C) contained 0.4 mM heterocyclic base, 0.4 mM PRPP, 0 to 5 mM MgCl2, 1.25 μg of TthAPRT.

We conducted several reactions to test the applicability of different heterocyclic bases for nucleotide synthesis. The data on nucleotide synthesis using *Tth*APRT are shown in *Table 3*.

**Fig. 7:**
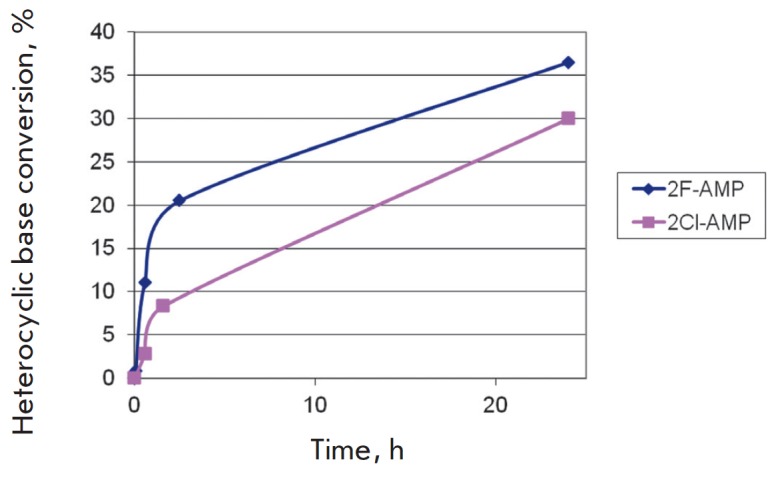
Cascade synthesis of 2Cl-AMP and 2F-AMP from appropriate heterocyclic bases and *D*-ribose. Reaction conditions: 0.5 mM *D*-ribose, 1 mM ATP, 0.4 mM heterocyclic base (2-chloro- or 2-fluoroadenine), 20 mM Tris-HCl (pH 8.0), 50 mM KCl, 1 mM MgCl_2_, 10 mM KH_2_PO_4_, 75°C, 0.3 μg of *Tsp*RK, 1.1 μg of *Tth*PRPPS1, 0.25 μg of *Tth*APRT in 250 μL of the reaction mixture.

The data in *Table 3* demonstrate that *Tth*APRT is specific to 6-aminopurines. 2-Chloroadenine, N1-methyladenine, and 2-methoxyadenine proved to be good substrates. No enzymatic activity was detected in reactions with hypoxanthine, guanine, N6-benzyladenine, aminobenzimidazole, and 1,2,4-triazole-3-carboxy-N-methylamide. The equilibrium in reactions with 2-chloroadenine was strongly shifted towards nucleotide formation (98% after 1 day). The reaction was conducted at 75 °C, which was preferable in the case of 2-chloroadenine because of its poor solubility. 2,6-Diaminopurine also was a substrate, while no product was detected in the reaction with its 7-deaza analogue, suggesting the need for a nitrogen atom at the seventh position of the purine.

**Fig. 8:**
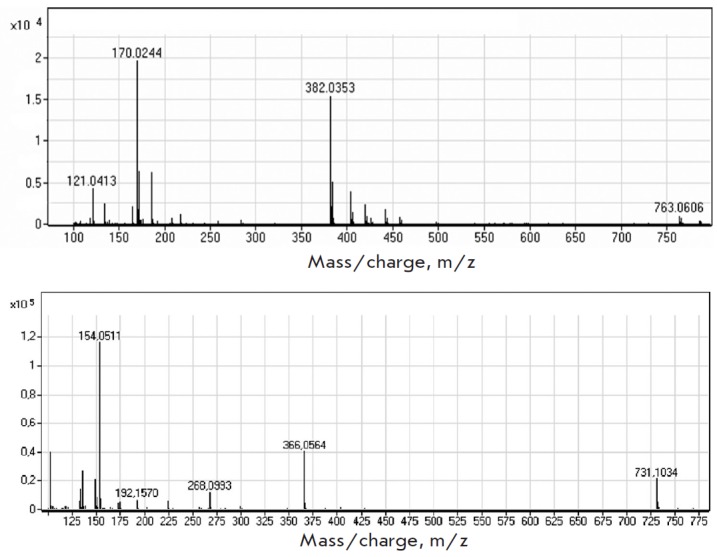
a) A mass spectrum of the 2Cl-AMP nucleotide produced by cascade synthesis:

After studying the substrate specificity of *Tth*APRT, we conducted a cascade synthesis of 2Cl-AMP and 2F-AMP from appropriate heterocyclic bases and *D*-ribose. The results are shown in *Fig. 7*. The process of cascade nucleotide synthesis was monitored by a liquid chromatography-mass spectrometry analysis of the reaction mixture; samples were taken at 1, 2, 24 h from the start of the process. *Figure 8* shows the mass spectra of the target products.

Therefore, by the synthesis of 2-chloro (fluoro)-adenosine monophosphate (2Cl-AMP and 2F-AMP), we demonstrated the relevance of a thermophilic enzymatic system for the production of bioactive nucleotides.

## CONCLUSION

Our findings indicate that a cascade of thermophilic enzymes of the nucleic acid metabolism (ribokinase; phosphoribosylpyrophosphate synthetase, and adenine phosphoribosyltransferase) can be used to produce modified nucleotides. This approach provides opportunities for the replacement of chemical methods of nucleotide synthesis with biocatalytic ones.

## Abbreviations

BSA: bovine serum albumin
IPTG: isopropyl-β-D-1-thiogalactopyranoside
PAAG: polyacrylamide gel
MSF: phenylmethylsulfonyl fluoride
PCR: polymerase chain reaction
APR-transferase (TthAPRT): adenine phosphoribosyltransferase from Thermus thermophilus
LB: Luria-Bertani medium
PRPP-synthetase (TthPRPPS): phosphoribosylpyrophosphate synthetase from Thermus thermophilus
RK (TspRK): ribokinase from Thermus sp.
2Cl-AMP: 2-chloroadenosine 5’-monophosphate
2F-AMP: 2-fluoroadenosine 5’-monophosphate
Pi: inorganic phosphate
PRPP: 5-phosphoribosyl-1-pyrophosphate
